# Estimating the distribution of dynamic invariants: illustrated with an application to human photo-plethysmographic time series

**DOI:** 10.1186/1753-4631-1-8

**Published:** 2007-07-23

**Authors:** Michael Small

**Affiliations:** 1 Department of Electronic and Information Engineering, Hong Kong Polytechnic University, Hung Hom, Kowloon, Hong Kong

## Abstract

Dynamic invariants are often estimated from experimental time series with the aim of differentiating between different physical states in the underlying system. The most popular schemes for estimating dynamic invariants are capable of estimating confidence intervals, however, such confidence intervals do not reflect variability in the underlying dynamics. We propose a surrogate based method to estimate the expected distribution of values under the null hypothesis that the underlying deterministic dynamics are stationary. We demonstrate the application of this method by considering four recordings of human pulse waveforms in differing physiological states and show that correlation dimension and entropy are insufficient to differentiate between these states. In contrast, algorithmic complexity can clearly differentiate between all four rhythms.

## 1. Background

Various dynamic invariants are often estimated from time series in a wide variety of scientific disciplines. It has long been known that these estimates (and in particular correlation dimension estimates) alone are not sufficient to differentiate between chaos and noise. Most notably, the method of surrogate data [1] was introduced in an attempt to reduce the rate of false positives during the hunt for physical examples of chaotic dynamics. Although it is not possible to find conclusive evidence of chaos through estimation of dynamic invariants, surrogate methods are often used to generate a distribution of statistic values (i.e. the estimates of the dynamic invariant) under the hypothesis of linear noise. In the most general form, the standard surrogate methods can generate the distribution of statistic values under the null hypothesis of a static monotonic nonlinear transformation of linearly filtered noise.

In this communication, we introduce a significant generalisation of a recent surrogate generation algorithm [2,3]. The *pseudo-periodic surrogate *(PPS) algorithm allows one to generate data consistent with the null hypothesis of a noise driven periodic orbit – provided the data exhibits pseudo-periodic dynamics. Previously, this algorithm has been applied to differentiate between a noisy limit cycle, and deterministic chaos. By modifying this algorithm and applying it to noisy time series data, we are able to generate surrogate time series that are independent trajectories of the same deterministic system, measured via the same imperfect observation function. That is, we assume that there is a deterministic dynamical system subject to additive independent and identically distributed (i.i.d.) observational noise. This ensemble of *attractor trajectory surrogates *(ATS) can then be used to estimate the distribution of statistic values for estimates of any statistic derived from these time series.

The statistics of greatest interest to us are dynamic invariants of the underlying attractor, and in particular correlation dimension and entropy estimates provided by the *Gaussian kernel algorithm *(GKA) [4,5]. Our choice of the GKA is entirely arbitrary, but based on our familiarity with this particular algorithm. True estimation of dynamic invariants from noisy data is a process fraught with difficulty, in this paper we are only concerned with estimating the distribution of estimates. To emphasise this point further we repeat out analysis with another quantity, Lempel-Ziv complexity [6], which *does not *constitute a dynamics invariant. Nonetheless, our algorithm provides a reliable estimate of the distribution of statistic values for this statistic as well.

An important application for the ATS technique is to determine whether dynamic invariants estimated from distinct time series are significantly different. The question this technique can address is whether (for example) a correlation dimension of 2.3 measured during normal electrocardiogram activity is really distinct from the correlation dimension of 2.4 measured during an episode of ventricular tachycardia [7,8]. Estimates of dynamic invariants (including the GKA [4,5]) often do come with confidence intervals. But these confidence intervals are only based on uncertainty in the least-mean-square fit, not the underlying dynamics. Conversely, it is standard practice to obtain a large number of representative time series for each (supposedly distinct) physical state, and compare the distribution of statistic values derived from these. But, this approach is not always feasible: in [7,8] for example, the problem is not merely that these physiological states are difficult and dangerous to replicate, but that inter-patient variability makes doing so infeasible.

In the remainder of this paper we describe the new ATS algorithm and demonstrate that it can be used to estimate the distribution of dynamic invariant estimates from a single time series of a known dynamical system (we demonstrate this with the Hénon map and the chaotic Rössler system). We then apply this same method to four recordings of human pulse waveforms, measured via photo-plethysmography [9,10]. Each of the four recordings correspond to a distinct physiological state. We compute correlation dimension and entropy using the GKA method and show that the expected distribution of correlation dimension and entropy estimates are insufficient to differentiate between these four physiological states. In contrast, we show that algorithmic complexity can clearly differentiate between all four rhythms.

In Section 2 we describe the algorithm we employ in this paper, and in Section 2.2 we demonstrate that, for suitable parameter values, this technique will preserve the deterministic dynamics of the underlying system. In Section 3 we present some numerical case studies and in Section 4 we finally present our conclusions.

## 2. Attractor trajectory surrogates

In the first part of this section we will review the PPS algorithm presented in [2] and describe the novel features of the ATS approach. In section 2.2 we examine the foundation of this technique's ability to preserve the underlying deterministic dynamics.

### 2.1 The algorithm

In what follows we assume that the measured scalar time series *x*_*t *_represents discretely sampled measurements of a deterministic dynamical system (possibly continuous) under the influence of observational noise. In other words, the dynamics are determined by a smooth manifold *M *and deterministic evolution operator *φ *: *M *→ *M*. The output of the evolution of an initial condition *m*_0 _∈ *M *under *φ *(i.e. *m*_*t *_= *φ*^*t*^(*m*_0_)) are observed via the differentiable function *h *: *M *→ **R**. Unfortuantely, experimental measurement is not perfect and the observed time series {*x*_*t*_} is subject to observational noise, hence, *x*_*t *_= *h*(*φ*^*t*^(*m*_0_)) + *ε*_*t *_where *ε*_*t *_~ N
 MathType@MTEF@5@5@+=feaafiart1ev1aaatCvAUfKttLearuWrP9MDH5MBPbIqV92AaeXatLxBI9gBamrtHrhAL1wy0L2yHvtyaeHbnfgDOvwBHrxAJfwnaebbnrfifHhDYfgasaacH8akY=wiFfYdH8Gipec8Eeeu0xXdbba9frFj0=OqFfea0dXdd9vqai=hGuQ8kuc9pgc9s8qqaq=dirpe0xb9q8qiLsFr0=vr0=vr0dc8meaabaqaciaacaGaaeqabaWaaeGaeaaakeaaimaacqWFneVtaaa@383B@ is drawn from some stationary noise distribution N
 MathType@MTEF@5@5@+=feaafiart1ev1aaatCvAUfKttLearuWrP9MDH5MBPbIqV92AaeXatLxBI9gBamrtHrhAL1wy0L2yHvtyaeHbnfgDOvwBHrxAJfwnaebbnrfifHhDYfgasaacH8akY=wiFfYdH8Gipec8Eeeu0xXdbba9frFj0=OqFfea0dXdd9vqai=hGuQ8kuc9pgc9s8qqaq=dirpe0xb9q8qiLsFr0=vr0=vr0dc8meaabaqaciaacaGaaeqabaWaaeGaeaaakeaaimaacqWFneVtaaa@383B@. For the case of dynamic noise, the situation is complicated further as the evolution of *m*_*t *_is governed by *m*_*t*+1 _= *φ*(*m*_*t*_) + *ξ*_*t *_where *ξ*_*t *_is stochastic.

The ATS algorithm may now be described as follows. Embed the observed scalar time series {*x*_*t*_} to obtain a vector time series {*z*_*t*_}, *z*_*t *_∈ **R**^*d*^, of *N *observations. The choice of embedding is arbitrary, but has been adequately discussed in the literature (there are numerous works in this field, [11] for example, provides references to several of them). We assume that the embedding is such that there exists a continuously differentiable map Ξ : *M *→ **R**^*d *^between the underlying manifold *M *and the embedding space **R**^*d *^such that both Ξ and *D*Ξ are one-to-one. Under these conditions, the dynamics of (*φ*, *M*) and the evolution of *z*_*t *_= Ξ(*m*_*t*_) ∈ **R**^*d *^are considered to be equivalent. From the embedded time series, the surrogate is obtained as follows. Choose an initial condition, *w*_1 _∈ {*z*_*j*_|*j *= 1, ..., *N*}. Then, at each step *i*, choose the successor to *w*_*i *_with probability

P(wi+1=zj+1)∝exp⁡−‖wi−zj‖ρ
 MathType@MTEF@5@5@+=feaafiart1ev1aaatCvAUfKttLearuWrP9MDH5MBPbIqV92AaeXatLxBI9gBaebbnrfifHhDYfgasaacH8akY=wiFfYdH8Gipec8Eeeu0xXdbba9frFj0=OqFfea0dXdd9vqai=hGuQ8kuc9pgc9s8qqaq=dirpe0xb9q8qiLsFr0=vr0=vr0dc8meaabaqaciaacaGaaeqabaqabeGadaaakeaacqWGqbaucqGGOaakcqWG3bWDdaWgaaWcbaGaemyAaKMaey4kaSIaeGymaedabeaakiabg2da9iabdQha6naaBaaaleaacqWGQbGAcqGHRaWkcqaIXaqmaeqaaOGaeiykaKIaeyyhIuRagiyzauMaeiiEaGNaeiiCaa3aaSaaaeaacqGHsisldaqbdaqaaiabdEha3naaBaaaleaacqWGPbqAaeqaaOGaeyOeI0IaemOEaO3aaSbaaSqaaiabdQgaQbqabaaakiaawMa7caGLkWoaaeaaiiGacqWFbpGCaaaaaa@4CED@

where the *noise radius ρ *is an as-yet unspecified constant. That is, the successor of *w*_*i*_, *w*_*i*+1 _is chosen to be the point *z*_*j*+1 _with probability proportional to exp⁡−‖wi−zj‖ρ
 MathType@MTEF@5@5@+=feaafiart1ev1aaatCvAUfKttLearuWrP9MDH5MBPbIqV92AaeXatLxBI9gBaebbnrfifHhDYfgasaacH8akY=wiFfYdH8Gipec8Eeeu0xXdbba9frFj0=OqFfea0dXdd9vqai=hGuQ8kuc9pgc9s8qqaq=dirpe0xb9q8qiLsFr0=vr0=vr0dc8meaabaqaciaacaGaaeqabaqabeGadaaakeaacyGGLbqzcqGG4baEcqGGWbaCdaWcaaqaaiabgkHiTmaafmaabaGaem4DaC3aaSbaaSqaaiabdMgaPbqabaGccqGHsislcqWG6bGEdaWgaaWcbaGaemOAaOgabeaaaOGaayzcSlaawQa7aaqaaGGaciab=f8aYbaaaaa@3DD0@, where *z*_*j *_is the antecedent of *z*_*j*+1_. In other words, the successor to *w*_*i *_is the successor of a randomly chosen neighbour of *w*_*i*_. Equation (1) may then be written as

P(wi+1=zj+1)=Pi,j∑k=1NPi,k
 MathType@MTEF@5@5@+=feaafiart1ev1aaatCvAUfKttLearuWrP9MDH5MBPbIqV92AaeXatLxBI9gBaebbnrfifHhDYfgasaacH8akY=wiFfYdH8Gipec8Eeeu0xXdbba9frFj0=OqFfea0dXdd9vqai=hGuQ8kuc9pgc9s8qqaq=dirpe0xb9q8qiLsFr0=vr0=vr0dc8meaabaqaciaacaGaaeqabaqabeGadaaakeaacqWGqbaucqGGOaakcqWG3bWDdaWgaaWcbaGaemyAaKMaey4kaSIaeGymaedabeaakiabg2da9iabdQha6naaBaaaleaacqWGQbGAcqGHRaWkcqaIXaqmaeqaaOGaeiykaKIaeyypa0ZaaSaaaeaacqWGqbaudaWgaaWcbaGaemyAaKMaeiilaWIaemOAaOgabeaaaOqaamaaqadabaGaemiuaa1aaSbaaSqaaiabdMgaPjabcYcaSiabdUgaRbqabaaabaGaem4AaSMaeyypa0JaeGymaedabaGaemOta4eaniabggHiLdaaaaaa@4BB6@

where Pi,k=exp⁡−‖wi−zj‖ρ
 MathType@MTEF@5@5@+=feaafiart1ev1aaatCvAUfKttLearuWrP9MDH5MBPbIqV92AaeXatLxBI9gBaebbnrfifHhDYfgasaacH8akY=wiFfYdH8Gipec8Eeeu0xXdbba9frFj0=OqFfea0dXdd9vqai=hGuQ8kuc9pgc9s8qqaq=dirpe0xb9q8qiLsFr0=vr0=vr0dc8meaabaqaciaacaGaaeqabaqabeGadaaakeaacqWGqbaudaWgaaWcbaGaemyAaKMaeiilaWIaem4AaSgabeaakiabg2da9iGbcwgaLjabcIha4jabcchaWnaalaaabaGaeyOeI0YaauWaaeaacqWG3bWDdaWgaaWcbaGaemyAaKgabeaakiabgkHiTiabdQha6naaBaaaleaacqWGQbGAaeqaaaGccaGLjWUaayPcSdaabaacciGae8xWdihaaaaa@43CF@ (and similarly, Pi,j=exp⁡−‖wi−zj‖ρ
 MathType@MTEF@5@5@+=feaafiart1ev1aaatCvAUfKttLearuWrP9MDH5MBPbIqV92AaeXatLxBI9gBaebbnrfifHhDYfgasaacH8akY=wiFfYdH8Gipec8Eeeu0xXdbba9frFj0=OqFfea0dXdd9vqai=hGuQ8kuc9pgc9s8qqaq=dirpe0xb9q8qiLsFr0=vr0=vr0dc8meaabaqaciaacaGaaeqabaqabeGadaaakeaacqWGqbaudaWgaaWcbaGaemyAaKMaeiilaWIaemOAaOgabeaakiabg2da9iGbcwgaLjabcIha4jabcchaWnaalaaabaGaeyOeI0YaauWaaeaacqWG3bWDdaWgaaWcbaGaemyAaKgabeaakiabgkHiTiabdQha6naaBaaaleaacqWGQbGAaeqaaaGccaGLjWUaayPcSdaabaacciGae8xWdihaaaaa@43CD@). Finally, from the vector time series {*w*_*i*_} the ATS {*s*_*i*_} is obtained by projecting *w*_*i *_onto [1 0 0 0 ⋯ 0] ∈ **R**^*d *^(the first coordinate). Hence

*s*_*t *_= *w*_*t*_·[1 0 0 0 ⋯ 0]

In [2,3] this algorithm was shown to be capable of differentiating between deterministic chaos and a noisy periodic orbit. In the context of the current communication we assume that {*x*_*t*_} is contaminated by additive (but possibly dynamic) noise and we choose the noise radius *ρ *such that the observed noise is replaced by an independent realisation of the same noise process. Furthermore, we assume that the deterministic dynamics are preserved by suitable choice of embedding parameters. Under these two assumptions, {*z*_*t*_} and {*w*_*t*_} have the same invariant density and {*x*_*t*_} and {*s*_*t*_} are therefore (noisy) realisation of the same dynamical system with (for suitable choice of *ρ*) the same noise distribution. We illustrate this more precisely in the following section.

### 2.2 Invariance

As in [2,3] the problem remains the correct choice of *ρ*. This is the major difference between the ATS described here and the PPS of [2,3]. However, since the null hypothesis we wish to address is different from (and more general than) that of the PPS, choice of *ρ *for the ATS is less restrictive. For *t *= *T *given, one can compute *P*(*w*_*t*+1 _≠ *z*_*i*+1 _∧ ||*w*_*t *_- *z*_*i*_|| = 0|*t *= *T*) directly from the data by applying (1) to the embedded time series(we use the symbol ∧ here in the usual manner to denote logical conjunction). Assuming the process is ergodic (that is, ergodic with respect to the standard measure – this assumption is sufficient rather than necessary) one can then sum

P(wi+1≠zj+1∧‖wi−zj‖=0)=1N∑T=1NP(wi+1≠zj+1∧‖wi−zj‖=0|j=T)
 MathType@MTEF@5@5@+=feaafiart1ev1aaatCvAUfKttLearuWrP9MDH5MBPbIqV92AaeXatLxBI9gBaebbnrfifHhDYfgasaacH8akY=wiFfYdH8Gipec8Eeeu0xXdbba9frFj0=OqFfea0dXdd9vqai=hGuQ8kuc9pgc9s8qqaq=dirpe0xb9q8qiLsFr0=vr0=vr0dc8meaabaqaciaacaGaaeqabaqabeGadaaakeaafaqaaeGabaaabaGaemiuaaLaeiikaGIaem4DaC3aaSbaaSqaaiabdMgaPjabgUcaRiabigdaXaqabaGccqGHGjsUcqWG6bGEdaWgaaWcbaGaemOAaOMaey4kaSIaeGymaedabeaakiabgEIizpaafmaabaGaem4DaC3aaSbaaSqaaiabdMgaPbqabaGccqGHsislcqWG6bGEdaWgaaWcbaGaemOAaOgabeaaaOGaayzcSlaawQa7aiabg2da9iabicdaWiabcMcaPiabg2da9aqaceaaO=VaaCzcamaalaaabaGaeGymaedabaGaemOta4eaamaaqahabaGaemiuaaLaeiikaGIaem4DaC3aaSbaaSqaaiabdMgaPjabgUcaRiabigdaXaqabaGccqGHGjsUcqWG6bGEdaWgaaWcbaGaemOAaOMaey4kaSIaeGymaedabeaakiabgEIizpaaeiaabaWaauWaaeaacqWG3bWDdaWgaaWcbaGaemyAaKgabeaakiabgkHiTiabdQha6naaBaaaleaacqWGQbGAaeqaaaGccaGLjWUaayPcSdGaeyypa0JaeGimaadacaGLiWoacqWGQbGAcqGH9aqpcqWGubavcqGGPaqkaSqaaiabdsfaujabg2da9iabigdaXaqaaiabd6eaobqdcqGHris5aaaaaaa@767F@

to get the probability of a temporal discontinuity in the surrogate at any time instant. By temporal discontinuity we mean that *w*_*i *_= *z*_*j *_but *w*_*i*+1 _≠ *z*_*j*+1_. That is, a point where the surrogate trajectory does not exactly follow the data. There is a one-to-one correspondence between a value *p *= *P*(*w*_*t*+1 _≠ *z*_*i*+1 _∧ ||*w*_*t *_- *z*_*i*_|| = 0) and *ρ*, and we choose to implement (1) for a particular value of *p *(i.e. a particular transition probability) rather than a specific noise level *ρ*. In what follows we find that studying intermediate values of *p *(*p *~ 0.1) is sufficient. For *p *∈ [0.1, 0.8] the qualitative behaviour over the corresponding narrow range of *ρ *is uniform. We choose to illustrate with *p *= 0.1, but the results for other choices are similar. Of course, for *p *→ 1 or *p *→ 0 the algorithm will not work well.

Now, suppose that the embedding parameters *τ *and *d*_*e *_have been selected correctly and the noise in the data is not too large, then the transformation *x*_*t *_↦ *z*_*t *_dictated by these parameters is an embedding. That is, the operator Ξ : *M *→ **R**^*d *^with Ξ(*m*_*t*_) = *z*_*t *_(in the absence of noise) and its derivative *D*Ξ are both one-to-one. Hence, the dynamic evolution of zj∈Rde
 MathType@MTEF@5@5@+=feaafiart1ev1aaatCvAUfKttLearuWrP9MDH5MBPbIqV92AaeXatLxBI9gBaebbnrfifHhDYfgasaacH8akY=wiFfYdH8Gipec8Eeeu0xXdbba9frFj0=OqFfea0dXdd9vqai=hGuQ8kuc9pgc9s8qqaq=dirpe0xb9q8qiLsFr0=vr0=vr0dc8meaabaqaciaacaGaaeqabaqabeGadaaakeaacqWG6bGEdaWgaaWcbaGaemOAaOgabeaakiabgIGioJqabiab=jfasnaaCaaaleqabaGaemizaq2aaSbaaWqaaiabdwgaLbqabaaaaaaa@3571@ can be represented by

*z*_*j*+1 _= Φ(*z*_*j*_) + *e*_*j*_

where Φ(·) is diffeomorphic to the true evolution operator (i.e. Φ = Ξ_°_*φ*_°_Ξ^-1 ^where *φ *: *M *→ *M *is the underlying evolution operator, defined earlier) and *e*_*j *_are uncorrelated noise vectors (corresponding to the terms *ε*_*t *_and possibly *ξ*_*t *_described earlier). Now we consider the process of constructing a surrogate. Let {wi}i=1N
 MathType@MTEF@5@5@+=feaafiart1ev1aaatCvAUfKttLearuWrP9MDH5MBPbIqV92AaeXatLxBI9gBaebbnrfifHhDYfgasaacH8akY=wiFfYdH8Gipec8Eeeu0xXdbba9frFj0=OqFfea0dXdd9vqai=hGuQ8kuc9pgc9s8qqaq=dirpe0xb9q8qiLsFr0=vr0=vr0dc8meaabaqaciaacaGaaeqabaqabeGadaaakeaadaGadeqaaiabdEha3naaBaaaleaacqWGPbqAaeqaaaGccaGL7bGaayzFaaWaa0baaSqaaiabdMgaPjabg2da9iabigdaXaqaaiabd6eaobaaaaa@3689@ denote the surrogate vector time series of length *N*. Clearly, setting *w*_1 _= *z*_*k *_for some randomly chosen *k *is simply some new initial condition. Now, *w*_*i*+1 _= *z*_*j*+1 _where *j *is chosen randomly from a distribution such that ||*w*_*i *_- *z*_*j*_|| is small. Let *ε*_*i *_= *z*_*j *_- *w*_*i *_corresponding to the small (random) perturbation introduced by selection according to (1), then

*w*_*i*+1 _= Φ(*w*_*i *_+ *ε*_*i*_) + *e*_*j*_.

Note that, *ε*_*j *_is the perturbation introduced in taking *z*_*j*_'s successor to be the successor of *w*_*i *_(it is a *dynamic noise *term, and it is a perturbation introduced by the ATS method). Conversely, *e*_*j *_is the dynamic error in applying Φ (this term is inherent to the data, and to our model of the data). By taking *n*-th iterates of (4) and (5) we see that the two noise terms *e*_*j *_and *ε*_*i*+1 _will combine. In other words, from (5) we get

*w*_*i*+2 _= Φ(Φ(*w*_*i *_+ *ε*_*i*_) + *e*_*j *_+ *ε*_*i*+1_) + *e*_*j*+1_,

and so on. Suppose that *e*_*j *_~ D
 MathType@MTEF@5@5@+=feaafiart1ev1aaatCvAUfKttLearuWrP9MDH5MBPbIqV92AaeXatLxBI9gBamrtHrhAL1wy0L2yHvtyaeHbnfgDOvwBHrxAJfwnaebbnrfifHhDYfgasaacH8akY=wiFfYdH8Gipec8Eeeu0xXdbba9frFj0=OqFfea0dXdd9vqai=hGuQ8kuc9pgc9s8qqaq=dirpe0xb9q8qiLsFr0=vr0=vr0dc8meaabaqaciaacaGaaeqabaWaaeGaeaaakeaaimaacqWFdepraaa@3827@ where D
 MathType@MTEF@5@5@+=feaafiart1ev1aaatCvAUfKttLearuWrP9MDH5MBPbIqV92AaeXatLxBI9gBamrtHrhAL1wy0L2yHvtyaeHbnfgDOvwBHrxAJfwnaebbnrfifHhDYfgasaacH8akY=wiFfYdH8Gipec8Eeeu0xXdbba9frFj0=OqFfea0dXdd9vqai=hGuQ8kuc9pgc9s8qqaq=dirpe0xb9q8qiLsFr0=vr0=vr0dc8meaabaqaciaacaGaaeqabaWaaeGaeaaakeaaimaacqWFdepraaa@3827@ is some noise distribution. Then, for the surrogates {*s*_*t*_} to be a new realisation of the system that generated {*x*_*t*_} we require that *e*_*j *_+ *ε*_*i*+1 _~ D
 MathType@MTEF@5@5@+=feaafiart1ev1aaatCvAUfKttLearuWrP9MDH5MBPbIqV92AaeXatLxBI9gBamrtHrhAL1wy0L2yHvtyaeHbnfgDOvwBHrxAJfwnaebbnrfifHhDYfgasaacH8akY=wiFfYdH8Gipec8Eeeu0xXdbba9frFj0=OqFfea0dXdd9vqai=hGuQ8kuc9pgc9s8qqaq=dirpe0xb9q8qiLsFr0=vr0=vr0dc8meaabaqaciaacaGaaeqabaWaaeGaeaaakeaaimaacqWFdepraaa@3827@. But this is equivalent to the condition that *z*_*j *_- *w*_*i *_~ *k*D
 MathType@MTEF@5@5@+=feaafiart1ev1aaatCvAUfKttLearuWrP9MDH5MBPbIqV92AaeXatLxBI9gBamrtHrhAL1wy0L2yHvtyaeHbnfgDOvwBHrxAJfwnaebbnrfifHhDYfgasaacH8akY=wiFfYdH8Gipec8Eeeu0xXdbba9frFj0=OqFfea0dXdd9vqai=hGuQ8kuc9pgc9s8qqaq=dirpe0xb9q8qiLsFr0=vr0=vr0dc8meaabaqaciaacaGaaeqabaWaaeGaeaaakeaaimaacqWFdepraaa@3827@ for sufficiently small *k*. Hence, the critical issue is the choice of *ρ *such that the these two noise terms are drawn from the same distribution and that therefore the surrogate dynamic (5) is equivalent to (4). This requires sufficient data, ergodicity, and *ρ *small enough. Note that, as *ρ *becomes smaller and the surrogate data become more like realisations of the same system, we also see less randomisation. This is a natural and unavoidable tradeoff.

## 3. Results

The following subsections present the application of this method for data generated from the Hénon map (section 3.1), the Rössler system (section 3.2) and experimental measurements of human pulse pressure waves (section 3.3).

### 3.1 The Hénon map

One potential difficulty of this method is that the stretching-and-folding characteristic of Smale horseshoe type chaos could easily destroy the dynamics of (5) and therefore produce surrogate trajectories that short-cut across the attractor. Although we can see from equations (5) and (6) that for sufficiently small perturbations this will never be the case, we would like to test this possibility in practise. For this purpose we apply the method described in the previous section to the extremely well studied Hénon map: one of the archetypes of Smale horseshoe chaos.

**Figure 1:**
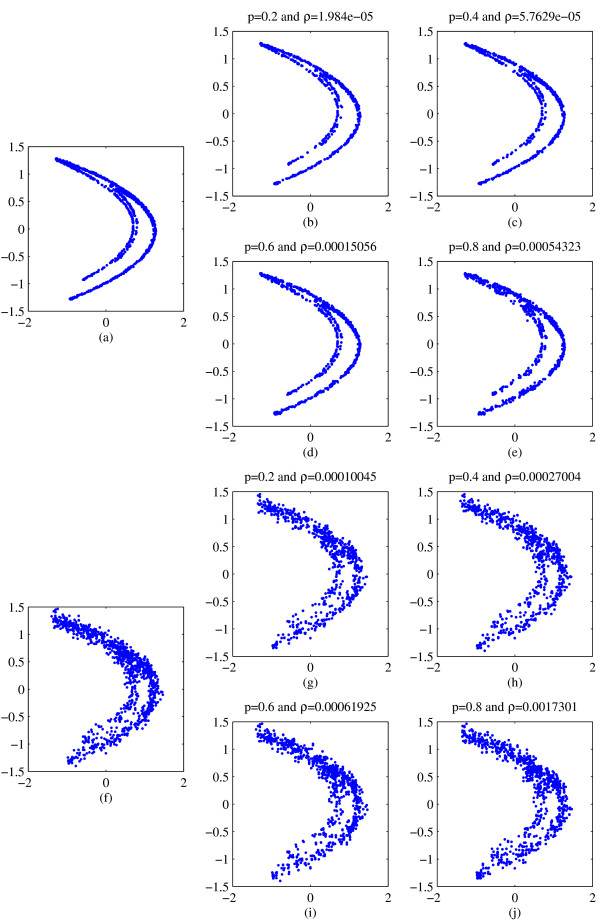
**Sample reconstructed attractors for data and surrogates of the Hénon map**. Panels (a) and (f) are embedded time series data from the *x*-component of the Hénon system with the addition of 1% and 10% observational noise (respectively). The remaining panels are representative ATS time series. Panels (b), (c), (d) and (e) are surrogates for panel (a), and Panels (g), (h), (i) and (j) are for panel (f). Each surrogate is computed with a different level of transition probability *P*. In panels (b) and (g), *p *= 0.2; in panels (c) and (h), *p *= 0.4; in panels (d) and (i), *p *= 0.6; and, in panels (e) and (j), *p *= 0.8. In each case the attractors reconstructed from the surrogates have the same qualitative features as that of the data – with the possible exception of panel (e). The likely reason for this noted exception is the relatively high transition probability (*p *= 0.8) and the relatively low noise level (1%). Of course, for smaller values of *p *(i.e. *p *= 0.1) the similarity is even more striking.

**Figure 2:**
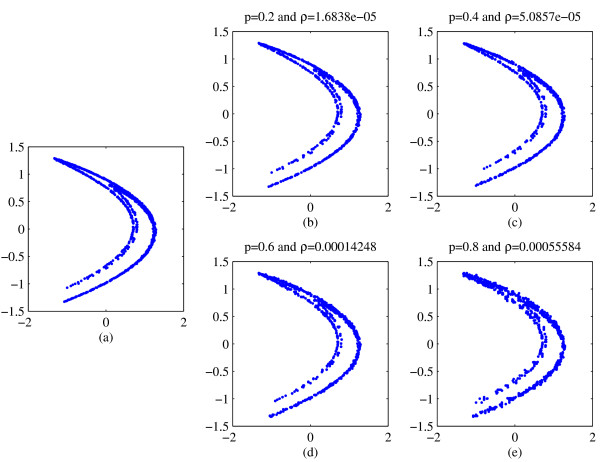
**Sample reconstructed attractors for data andsurrogates of the Hénon map**. Panel (a) is an embedded time series data from the *x*-component of the Hénon system with the addition of 1% *dynamic *noise. The remaining panels are representative ATS time series. Each surrogate is computed with a different level of transition probability *P*. In panel (b) *p *= 0.2; in panel (c) *p *= 0.4; in panel (d) *p *= 0.6; and, in panels (e) *p *= 0.8. In each case the attractors reconstructed from the surrogates have the same qualitative features as that of the data.

Figure 1 illustrate typical ATS calculations for this data set. Using short (1000 point) sections of the Hénon system, with the addition of observational noise (the Figures show 1% and 10% noise levels), we computed typical ATS data for different values of transition probability *p*. We find that in almost all cases (see Figure 1) the results for the ATS data agree qualitatively with the data. Comparison of estimated dynamic invariant (results omitted) confirm this. In all cases, for moderate range of *p *(i.e. *p *neither approaching 0 or 1) and moderate observational noise, we find data and surrogate agree closely. When this same computation was repeated for *dynamic *noise, we found data and surrogates to be similarly indistinguishable (see Figure 2): except for the case of large *p *and small noise (in this case, 1% dynamic noise and *p *= 0.8). Note that, for the Hénon map larger values of dynamic noise will actually force the system into an unstable régime.

### 3.2 The Rössler system

We now demonstrate the applicability of this method for a more realistic example: noisy time series data simulated from the Rössler differential equations (during "broad-band" chaos). We integrated (one thousand points with a time step of 0.2) the Rössler equations both with and without multidimensional dynamic noise at 5% of the standard deviation of the data. As far as possible, we generated realisations of the Rössler system that superficially resemble the physiological data of 3.3. The purpose of this is to provide a more realistic test of our method. We then studied the *x*-component after the addition of 5% observational noise. We selected embedding parameters using the standard methods (yielding *d*_*e *_= 3 and *τ *= 8) and then computed ATS surrogates for various exchange probabilities *p *= 0.05, 0.1, 0.15, ..., 0.95.

**Figure 3:**
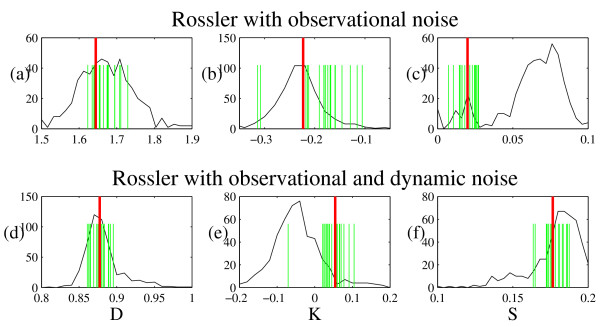
**Distribution of statistics *D*, *K *and *S *for short and noisy realisations of the Rössler system**. The histogram shows the distribution of statistic estimates (*D*, *K *and *S*) for 500 ATS time series generated from a 1000 point realisation of the Rössler system. The tall vertical line on each plot is the comparable value for the data and the shorter vertical lines indicate 20 independent realisations of the same process. The top row of figures depicts results for the Rössler system with observational noise only, the bottom row of figures has both observational and dynamic noise. Panels (a) and (d) show correlation dimension estimates, (b) and (e) are entropy, and (c) and (f) are noise level.

For the data set and each ensemble of surrogates we then estimated correlation dimension *D*, entropy *K *and noise level *S *using the GKA algorithm [4,5]. The GKA embedding used embedding dimension *m *= 2, 3, ..., 10 and embedding lag of 1. It is important to note that, a correlation dimension estimate is not the same thing as the actual correlation dimension. In particular, this algorithm estimates correlation dimension and noise level simultaneously (as well as entropy). A lower correlation dimension (associated with the presumed determinism in the system) is accompanied by an increase in the estimated noise level. That is, the estimated dimension can be lower because the algorithm is attributing more of the variation in the data to noise, and therefore estimating a higher noise level (and hence, in some case, the correlation dimension falls below 1). Similarly, the fact that the entropy is negative in the first case is associated with the system noise. Nonetheless, we are using these numbers only as measures, that is, as test statistics. Figure 3 depicts the results when the GKA is applied with embedding dimension *m *= 4 and the exchange probability is *p *= 0.1. Other values of *m *gave equivalent results, as did various values of *p *in the range [0.1, 0.8].

For such moderate *p *we found that the estimate of noise *S *from the GKA algorithm coincided for data and surrogates, but this was often not the case for more extreme values of *p*. This estimate of signal noise content is therefore a strong test of the accuracy of the dynamics reproduced by the ATS time series. One expects this to be the case as noise level is precisely the parameter upon which the ATS method depends. Furthermore to confirm the spread of the data we also estimated *D*, *K*, and *S *for 20 further realisations of the same Rössler system (with different initial conditions). In each case, as expected, the range of these values lies within the range predicted by the ATS scheme. We do see, for example, in Figure 3(c) that the range of noise level exhibited by the true Rössler system is not as expansive as that for the surrogates (to some extent, we can also observe the same problem with entropy in Figure 3(e)). This is due to the fact that the ATS method can be made to introduce more randomisation than absolutely necessary. By tuning down the randomisation we (obviously) will converge to the true data. By increasing the randomisation we cover an ever widening range, which will always include the true value. For large randomisation, and for statistics that are most sensitive to noise (in this case *K *and *S*) there may also be some bias – the observed difference in the means. Although it is desirable that both distributions coincide exactly, it is re-assuring (and sufficient) that the ATS distribution contains the true distribution.

### 3.3 Photo-plethysmographic recordings

**Figure 4:**
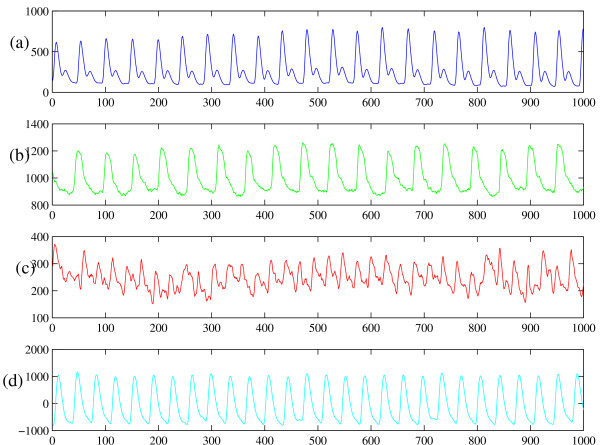
**Human pulse waveform recorded withphoto-plethysmography**. Four recordings of human pulse waveform (61 Hz) in four different physiological conditions. The four time series correspond to: (a) normal, (b) quasi-stable, (c) unstable, and (d) post-operative (stable).

We now consider the application of this method to photo-plethysmographic recordings of human pulse dynamics over a short time period (about 16.3 seconds). We have access to only a limited amount of data representative of each of four different dynamic regimes. In any case, we would expect the system dynamics to change if measured over a significantly longer time frame. The data collection and processing with the methods of nonlinear time series analysis are described in [9,10]. Previously, we have studied nonlinear determinism in cardiac dynamics measured with electrocardiogram (ECG) [7,8]. Although we do not consider ECG data here, this data would be another useful system to examine with these methods. Actually, the problem with ECG data is that we have too much data and it is therefore difficult to fairly select a "representative" small number of short time series. However, we intend to examine this data more carefully in the future. However, we do note in passing that both PPG and ECG are measures of cardiac activity and are therefore potentially equivalent [12,13]. The four data sets we examine in this communication are depicted in Figure 4.

**Figure 5:**
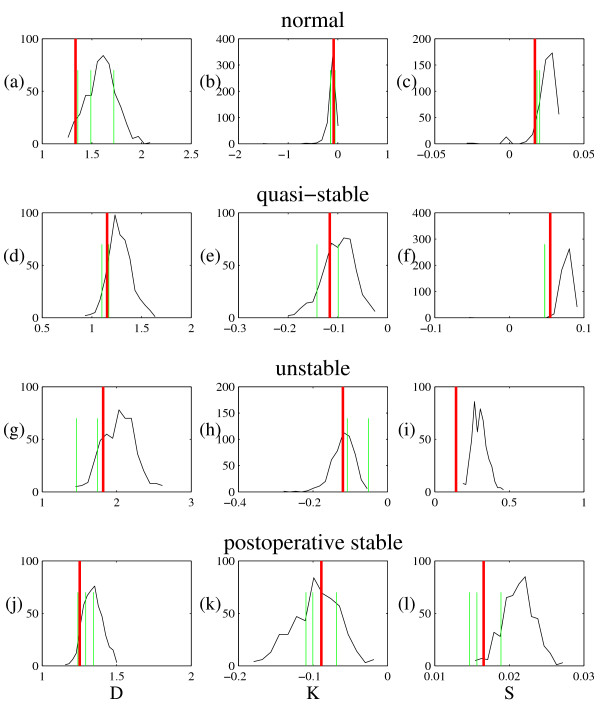
**Distribution of statistics *D*, *K *and *S *for human pulse waveforms**. The histogram shows the distribution of statistic estimates (*D*, *K *and *S*) for 500 ATS time series generated from each of the four time series depicted in figure 4. The taller vertical line on each plot is the comparable value for the shorter vertical lines are for the (limited) subsequent data recorded from each patient. In each case only two or three subsequent contiguous but non-overlapping time series were available. The figures are: (a) correlation dimension (*D*), (b) entropy (*K*), and (c) noise (*S*) for the normal rhythm; (d) *D*, (e) *K*, and (f) *S *for the quasi-stable rhythm; (g) *D*, (h) *K*, and (i) *S *for the unstable rhythm; and (j) *D*, (k) *K*, and (l) *S *for the post-operative stable rhythm.

For each data set we repeated the analysis described for the Rössler time series. Results for GKA embedding dimension *m *= 6 and *p *= 0.1 are depicted in Figure 5. As with the Rössler system, variation of the parameters *m *and *p *did not significantly change the results. We find that in every case (except for extreme values of *p*) the distribution of *D*, *K *and *S *estimated from the ATS data using the GKA included the true value. Most significantly, this indicates that the range of values of *p *is appropriate. Moreover, these results are consistent with the hypotheses that the noise is effectively additive and can be modelled with this simple scheme, and that the underlying deterministic dynamics can be approximated with a local constant modelling scheme.

We also estimated the statistics *D*, *K *and *S *for additional available data (subsequent, contiguous, but non-overlapping) from each of the four rhythms. This small amount of data afforded us two or three additional estimates of each statistic for each rhythm. For the unstable and quasi-stable rhythm we observed good agreement. For the stable (normal and post-operative) rhythms, this is not the case. On examination of the data we find that this result is to be expected. Both the stable rhythms undergo a change in amplitude and baseline subsequent to the end of the original 16 second recording, this non-stationarity is reflected in the results. This same non-stationarity has also been observed independently in Bhattacharya and co-workers [9,10].

**Figure 6:**
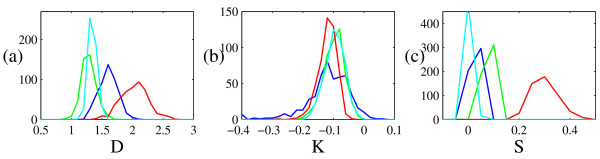
**Discriminating power of the statistics *D*, *K *and *S *for human pulse waveforms**. The distribution (a binned histogram) of statistic values estimated via the ATS method (as described in figure 5) for each of the four distinct physiological waveforms is shown. The four rhythms correspond to those in Figure 4. These figures show that correlation dimension alone is sufficient to differentiate between three of these four physiological states: on the left, "post-operative" and "quasi-stable" are indistinguishable, the correlation dimension for "normal" is bigger, and "unstable" is larger again We see that these three statistics are insufficient to differentiate between the "quasi-stable" and "post-operative" states, moreover, there is considerable overlap with the "normal" group.

We now return to the question that the ATS test was designed to address: can we differentiate between these four rhythms based on the GKA? Figure 6 provides the answer. In Figure 6 we see the estimated distribution of statistic values (*D*, *K *and *S*) for each of the four rhythms shown in figure 4. Clearly (and not surprisingly), the correlation dimension estimate and noise level of the unstable rhythm is significantly different from the other three rhythms.

**Figure 7:**
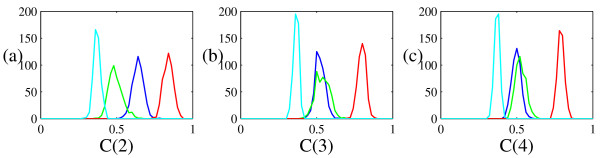
**Discriminating power of complexity for human pulse waveforms**. The distribution (a binned histogram) of statistic values estimated via the ATS method (as described in figure 5) for each of the four distinct physiological waveforms is shown. The four rhythms correspond to those in figure 4. These figures show that complexity with 2, 3, and 4, symbols (plots (a), (b), and (c), respectively) is sufficient to differentiate between at least three of these four physiological states. The lowest complexity corresponds to "post-operative" state, the next highest to "quasi-stable" followed by "healthy" and finally "unstable". As in figure 6 there is considerable overlap between the "normal" and "quasi-stable" samples. However, for complexity with a binary partition (panel (a)) the four rhythms do appear to be distinct.

Our analysis indicates that, contrary to what one may expect from individual measurements, the stable or "quasi-stable" rhythms cannot be properly distinguished based on these nonlinear statistics derived from the GKA. Moreover, we find that entropy estimated with the GKA algorithm *K *is of no use in differentiating between any of these four rhythms. Although it is not the purpose of this paper to provide a discriminating statistic for this data, it would be nice to do so. Therefore, in Figure 7 we repeat the calculation of surrogates and statistic distribution for the same data, but using algorithmic complexity (see [11] and the references therein) with binary, ternary, and quaternary encodings with equal likelihood for each symbol. Using this scheme it can be seen from Figure 7 that it is possible to distinguish, with a high level of certainty between three of these rhythms. Distinguishing between all four is also possible, with a small likelihood of error (see Figure 7(a)).

## 4. Conclusion

The results of this analysis are in general agreement with those presented in [9,10]. Independent linear surrogate analysis [1] has confirmed that each of these four rhythms is inconsistent with a monotonic nonlinear transformation of linearly filtered noise (these calculations are routine, and not presented in this paper). The only significant difference is that the correlation dimension estimates we present here are significantly lower than those in [9,10]. This is due to the different correlation dimension algorithm. Unlike the algorithm employed in [9,10], the GKA seperates the data into purely deterministic and stochastic components, and hence estimates both *D *and *S*. The correlation dimension estimated in [9,10] is the combined effect of both components of the GKA.

Although we have considered the specific application to human pulse dynamics, the algorithm we have proposed may be applied to a wide variety of problems. We have shown that provided time delay embedding parameters can be estimated adequately, and an appropriate value of the exchange probability is chosen, the ATS algorithm generates independent trajectories from the same dynamical system. When applied to data from the Rössler system we confirm this result, and we demonstrate its application to experimental data.

When the ATS algorithm is applied to generate independent realisation of a hypothesis test, one is able to construct a test for non-stationarity. If two data sets do not fit the same distribution of ATS data then they can not be said to be from the same deterministic dynamical system. Unfortunately, the converse is not always true and the power of the test depends on the choice of statistic. The utility of this technique as a test for stationarity remains uncertain.
